# Impact of Speller Size on a Visual P300 Brain-Computer Interface (BCI) System under Two Conditions of Constraint for Eye Movement

**DOI:** 10.1155/2019/7876248

**Published:** 2019-07-01

**Authors:** R. Ron-Angevin, L. Garcia, Á. Fernández-Rodríguez, J. Saracco, J. M. André, V. Lespinet-Najib

**Affiliations:** ^1^Departamento de Tecnología Electrónica, ETSI Telecomunicación, Universidad de Málaga, Málaga, Spain; ^2^IMS UMR 5218, CIH, ENSC, Bordeaux INP, Bordeaux, France; ^3^Univ. Bordeaux, CNRS, Bordeaux INP, IMB, UMR 5251, F-33400 Talence, France; ^4^INRIA, IMB, UMR 5251, F-33400 Talence, France

## Abstract

The vast majority of P300-based brain-computer interface (BCI) systems are based on the well-known P300 speller presented by Farwell and Donchin for communication purposes and an alternative to people with neuromuscular disabilities, such as impaired eye movement. The purpose of the present work is to study the effect of speller size on P300-based BCI usability, measured in terms of effectiveness, efficiency, and satisfaction under overt and covert attention conditions. To this end, twelve participants used three speller sizes under both attentional conditions to spell 12 symbols. The results indicated that the speller size had, in both attentional conditions, a significant influence on performance. In both conditions (covert and overt), the best performances were obtained with the small and medium speller sizes, both being the most effective. The speller size did not significantly affect workload on the three speller sizes. In contrast, covert attention condition produced very high workload due to the increased resources expended to complete the task. Regarding users' preferences, significant differences were obtained between speller sizes. The small speller size was considered as the most complex, the most stressful, the less comfortable, and the most tiring. The medium speller size was always considered in the medium rank, which is the speller size that was evaluated less frequently and, for each dimension, the worst one. In this sense, the medium and the large speller sizes were considered as the most satisfactory. Finally, the medium speller size was the one to which the three standard dimensions were collected: high effectiveness, high efficiency, and high satisfaction. This work demonstrates that the speller size is an important parameter to consider in improving the usability of P300 BCI for communication purposes. The obtained results showed that using the proposed medium speller size, performance and satisfaction could be improved.

## 1. Introduction

Evoked brain signals using different stimulus modalities can be employed to translate human intentions into external actions (motor outputs) through brain-computer interface (BCI) systems [1, 2]. These systems enable a nonmuscular channel of communication between the user and his environment, which could be especially useful for people diagnosed with severe motor disorders such as amyotrophic lateral sclerosis (ALS). So these systems are the only option that some patients have to communicate and get autonomy.

BCI systems based on electroencephalographic (EEG) signal recording are the most widely studied. In spite of existing different EEG-based BCI systems, a visual P300-based BCI speller is the main interface used for communication and control purposes and represents a reliable real option to supply the needs of people with neurological dysfunctionality [3, 4]. The P300 signal is a type of event-related potential (ERP) which is mostly recorded over the central and parietal regions. Specifically, it is a positive deflection of brain activity which occurs about 300 ms after an odd stimulus presentation.

There are several types of P300-based spellers, such as auditory, tactile, or visual. The main advantage of auditory and tactile modalities is that they do not depend on the ocular capacity (e.g., [5]). However, if the user retains some residual ocular mobility, the visual interfaces will show a better performance (see Rezeika et al. [6] for a review of P300-based spellers). The vast majority of P300-based BCI spellers studied and developed are based on the one proposed by Farwell and Donchin in 1988 [7]. Farwell and Donchin's speller consisted of a 6 × 6 matrix of characters. Its rows and columns are flashed (i.e., stimulated) pseudorandomly, one by one, while the user pays attention on his target element from the matrix. The stimulation of the user's target element represents the “rare event” of the oddball paradigm and elicits the P300. After a specified number of row and column stimulations, the computer identifies the matrix element to which the user is attending as the intersection of the row and column that elicits the largest P300, and this symbol is shown on the screen.

Given a number of studies which were carried out on both healthy subjects and patients affected by some motor disability, the effectiveness of the mentioned P300-based spellers is proved [4]. Overall, these studies agree on that the P300 speller gives an effective communication channel to those patients who have almost—or completely—lost the possibility to write or speak. However, as it is proposed in [8], the BCI spellers' usability is still needed to get better. The current definition of usability given by the International Organization for Standardization (ISO 9241-11) involves three measures: (i) effectiveness (i.e., accuracy and completeness of the system with which users achieve set goals), (ii) efficiency (i.e., resources expended to complete goals), and (iii) satisfaction (i.e., users' attitude to complete a given task) [9–11]. Froekjaer et al. came to the conclusion that these measures should be considered as independent usability aspects [12]. The efficiency and satisfaction could be basically measured through different subjective aspects: mental workload, fatigue, motivation, comfort, pleasure to use, and so on [13, 14].

The P300 signal amplitude and latency can be influenced by many factors, for instance, the mental fatigue after a long use [15, 16], the level of kept attention to a desired symbol [17], the user's motivation [15, 18], or the user's frustration (see [19] for a review). In this regard, the attention of researchers is increasingly focused on the effect on the user performance given several temporal and spatial aspects of the speller interfaces [20].

Although a vast majority of papers have focused on signal-processing algorithms in order to improve the performance of the P300 BCI system, there are several researches that have studied parameters which might have an influence on the user performance. Some of these parameters are the stimulus timing features [21–23], the effect of luminosity contrast [24], and the influence of interface colour contrast [25]. Regarding the effect of matrix configuration, the research is limited. Some studies have demonstrated how the user performance is affected by the matrix size. Specifically, Allison and Pineda [26] made a study where three matrix sizes (4 × 4, 8 × 8, and 12 × 12) were compared. The results indicated that larger P300 amplitudes were evoked by larger matrices and the user performance or preference was not significantly affected by the matrix size. On the contrary, a study where two different matrix sizes were compared (3 × 3 and 6 × 6) showed that the 3 × 3 matrix achieved higher accuracy, whereas the P300 amplitude was higher for the 6 × 6 matrix condition [22]. In both studies, symbol size was the same in different matrices, and thus, the larger matrices were presented larger on the monitor by only increasing the distance between symbols. As a result of these studies, some P300 BCI spellers used a reduced matrix to increase the writing speed [27]. Salvaris and Sepulveda studied the effects on classification of changes in the dimensions of the symbols, the distance between the symbols, and the background colours [28]. In this study, only two different values of each parameter were compared: small symbol size versus large symbol size, small intersymbol distance versus large intersymbol distance, and black background versus white background. The worst performance was obtained with the small symbol size.

There have been no studies related to the effect of speller size, apart from the mentioned studies about matrix size and symbol size. In [29], three different screen sizes were tested: a computer monitor, a global positioning system (GPS) screen, and a mobile phone screen. Nevertheless, no symbol size information was provided. According to the information provided about the screen resolution and the distance from the participants to each screen, the visual field for the computer monitor was 6.4°, for the GPS screen 3.7°, and for the mobile phone screen 3.56°, where two smallest screens had almost the same visual fields. However, since this study did not provide information regarding the speller size, it is unclear how their results are related to speller size. Actually, this study's main purpose was to assess BCI performance when these three specific screens were used but not to study the effect of screen size. To evaluate the effect of speller size, different visual fields should be proposed in terms of both symbol size and symbol distance, as they are crucial to confirm the proposals of different speller sizes.

Most of the P300 visual spellers are used in the overt attention mode, that is, allowing the subjects to fixate the target with their eyes. However, several studies have also proposed P300 visual speller usage in the covert attention mode [30–33] as an alternative communication aid for completely locked people. In this mode, subjects have to fixate the centre of the screen while paying attention to the target using visual periphery. Effectively, unfortunately, some of the potential users of a BCI speller, that is, ALS patients, could have impaired visual function, not allowing to gaze different targets. In our study, the covert attention mode has been employed to replicate the lack of ocular mobility that is suffered for some patients with severe motor disorders. Some of these proposed studies [30, 33] have clearly demonstrated that the performance of the classical speller in the covert attention condition considerably decreases compared to the overt attention condition. Degradation of spatial acuity in the peripheral vision is one of the effects that contribute to reduce this performance [31]. Human detail vision is limited to the fovea (centre), where visual acuity is 100%. As the distance from the fovea increases (eccentricity), the visual acuity drops rapidly to approximately 60% at 1° eccentricity, 50% at 2°, 30% at 7°, and 20% at 10° [34]. This degradation of visual acuity as a function of eccentricity should be taken into account in the design of BCI P300 speller in the covert attention condition. In a classical speller, one way to prevent the detrimental effects of declining visual acuity is to reduce the speller size. However, the symbol sizes would also reduce and be more difficult to distinguish. In this sense, it would also be interesting to study the effect of different speller sizes in the covert attention condition.

A published study by Brunner et al. [33] investigated the extent to which the performance of a classical P300 BCI speller depends on eye gaze. To this end, they evaluated the offline performance of 17 healthy subjects under overt and covert attention conditions. The obtained results showed a significant reduction in the classification accuracy in the covert attention condition compared to the overt attention condition. As it is mentioned in this paper, further studies are necessary to evaluate the effect of online feedback (online performance). On the contrary, it would be interesting to evaluate the workload required in both conditions.

The goal of this study was to explore the extent to which the performance of a classical P300 speller depends on speller size. The purpose was to better understand how easily a user can carry out the speller task comfortably and efficiently by analysing the same speller system type while using different BCI speller sizes. In this sense, we evaluated the usability of different speller sizes in terms of effectiveness, efficiency, and satisfaction [10, 35]. Effectively, the obtained performance is not a sufficient criterion to determine whether a user would want to use an interface. To this end, it is necessary to take into account these three parameters (effectiveness, efficiency, and satisfaction), making it possible not only to predict the user's intention [36] and the degree of acceptance of an interface [37, 38] but also to offer a better user experience [39].

## 2. Methods

### 2.1. Participants

Twelve French university students (seven males and five females; age range 19–25 years (20.6 ± 0.9 years)) participated in the present study (S1–S12), which consisted of six sessions, one for each speller size (i.e., small, medium, and large) and for each attentional condition (i.e., overt and covert). According to self-reports, all participants had no history of neurological or psychiatric illness and had normal or corrected to normal vision. Every participant gave informed consent through a protocol reviewed by the ENSC-IMS cognitive team. None of them had previous experience with BCI systems. The study was approved by the Ethics Committee of the University of Malaga and met the ethical standards of the Helsinki Declaration.

### 2.2. EEG Data and Processing

EEG recording and amplifying was through a 16-channel biosignal amplifier (g.BSamp, Guger Technologies) of gold electrodes. According to the 10/20 international system, the electrodes were placed at positions Fz, Cz, Pz, Oz, P3, P4, PO7, and PO8. The channels' reference was the right earlobe, and FPz was used as ground. Through the amplifier settings, the signal was bandpass filtered at 0.5 and 100 Hz, the notch filter (at 50 Hz) was on, and the sensitivity was 500 *µ*V. Next, the EEG data were digitized at a rate of 256 Hz by a 12-bit resolution NI-USB-6210 data acquisition card (National Instruments). Every aspect of EEG data recording and processing was controlled by the BCI2000 software [20].

### 2.3. BCI Speller

The BCI speller used was the classical Farwell and Donchin [7] speller, which consists of a 6 × 6 matrix of symbols (36 alphanumeric letters and numbers) arranged in rows and columns. The temporal parameter values for all the spellers were based on those used by Donchin et al. [40]. Specifically, each row and each column were intensified (i.e., flashed) pseudorandomly 10 times, and thus, each character was intensified 20 times. Both the stimulus presentation duration (i.e., the duration of each flash) and the interstimulus interval (ISI) pause between stimulus presentations were 125 ms. A pause of 6 sec was used following each sequence of flashes (i.e., pause between each character selection). This pause duration was selected to give the subject time to look for the new target character and gaze it. In the covert attention condition, subjects were also allowed to gaze the new target character during this pause. Considering these temporary parameter values, each symbol needed a time of 36 s to be selected (as it is 10 times the sum of the flash duration—125 ms—of six rows and six columns with an ISI of 125 ms, plus the 6 s after the sequences of flashes).

### 2.4. Speller Size

Three different speller sizes were proposed. The screen used to present the spellers was 17″ TFT with a refresh rate of 60 Hz and a resolution of 1440 × 900 px^2^. Each speller consisted of a 6 × 6 matrix of 36 characters which was centred on the screen.

- The speller size used in [30] was chosen as the largest size of the present study because it is frequently applied by other researchers (e.g., [33]). The matrix subtended ±6.98° of the visual field both horizontally and vertically. The size of each character was 1.12° *W* × 1.12° *H*, with the horizontal separation between columns being 1.46° *W* and the vertical separation between rows being 1.46° *H*.
- The smallest speller size was chosen according to what was reported by Salvaris and Sepulveda [28] as the smallest symbol size which could be used without loss of spelling performance. As the subjects were seated 1 m from the screen in their experiments and the smallest symbol size was 0.7 cm *W* × 0.8 cm *H* in [28], in terms of visual field, the symbol size was equivalent to 0.4° *W* × 0.45° *H*. To maintain the same characteristics regarding the size of different spellers, the symbol size used in our experiment should be the same in height and width. Finally, each character's size on the smallest speller was 0.4° *W* × 0.4° *H*. This small symbol size (0.4° *W* × 0.4° *H*) represents a reduction of 35.89% of the largest symbol size chosen (1.12° *W* × 1.12° *H*). In order to preserve the same proportions between speller sizes, the smallest speller subtended a visual field of ±2.51° both horizontally and vertically, and the horizontal and vertical separation between columns and rows, respectively, was 0.52° (i.e., 35.89% less compared to the largest size). With this size and according to the study in [33], in the cover attention condition, the visual acuity would drop to approximately 50% for targets located further away from the centre.
- The medium size was selected calculating the middle value between the large and small speller sizes. Then, the matrix subtended ±4.75° of the visual field both horizontally and vertically, the intermediate symbol size was 0.75° *W* × 0.75° *H*, and the horizontal and the vertical separation between columns and rows, respectively, was 1°.

**Figure 1:**
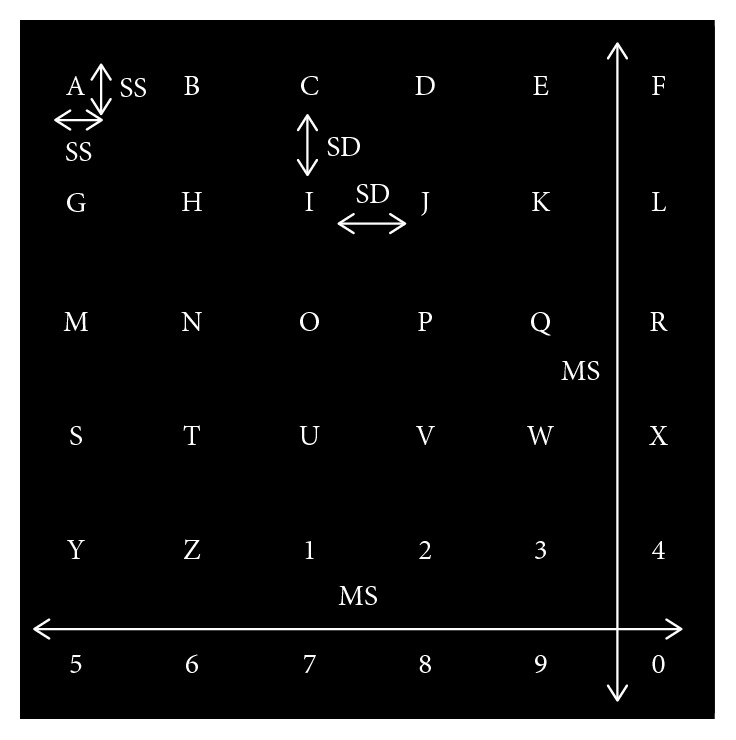
Size metrics determined for the visual protocol.

Taking into account that subjects were situated at a distance of about 60 cm from the screen, the measures of each speller are presented in Figure 1 and Table 1.

### 2.5. Experimental Design

The three different speller sizes were tested by every participant following a within-subject design. Thus, the experiment consisted of three sessions, wherein each of them tested one speller. Sessions were carried out on different days, and the time interval between each session was between three and five days (both included). The order in which the spellers were assessed was counterbalanced over participants to control for the potential effects of experience. In order to measure eye gaze, an eye tracker (Tobii X1, Tobii Technology) was mounted under the screen. All sessions took place inside an isolated experimental room.

### 2.6. Task and Procedure

Prior to the experiment, instructions were given in written and verbal forms. All participants were seated at a distance of approximately 60 cm from the screen, which is the optimal operational range for the eye tracker (60 ± 10 cm). Each subject used the three speller sizes (small, medium, and large) under two attentional conditions (overt and covert attention). In the overt attention condition, the participant was asked to gaze at the target (i.e., the desired symbol that should be written). In the covert attention condition, the subject had to gaze only at a yellow dot located in the centre of the screen while counting the intensification of the desired character. Likewise, each session consisted of two tasks for each condition: a calibration task and an online task. Before the calibration task of the BCI speller, the eye tracker was calibrated.

Before the calibration task started, the participants were informed that he/she would see 10 random short intensifications (i.e., flashes) of rows and columns. Each time all the rows and columns were flashed 10 times, a sequence was completed. As every row and column flashed 10 times (i.e., 10 sequences), each character was flashed 20 times. The mental task for participants to type a letter or a number was to mentally count every time that his/her desired symbol was flashed before the 6 sec pause. During the calibration task, the participants did not receive any feedback and were asked to focus consecutively on 16 characters to spell three French words and a number, all of them with four characters (four runs). The spelled words were “LUNE,” “FEUX,” and “KILO” and the number was “2015.” While they were doing the task, their EEG data were recorded in order to analyse them afterwards. The calibration task took approximately 10 minutes. At the end of the calibration task, a stepwise linear discriminant analysis of the last three runs was performed to get the weights of the P300 classifier.

**Figure 2:**
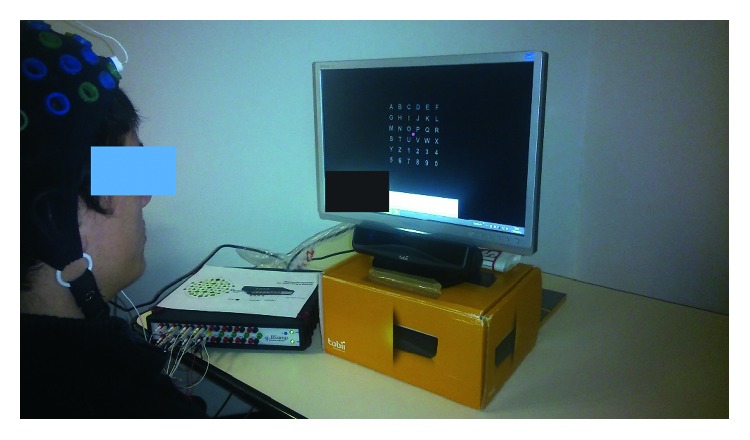
Subject during the experimental test. A Tobii eye tracker is mounted under the screen.

**Figure 3:**
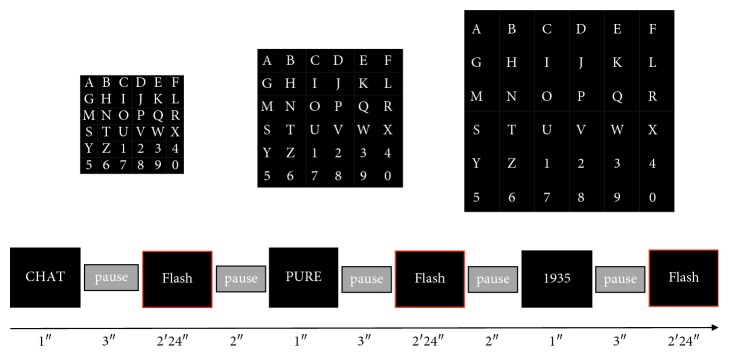
Temporal sequence employed on the online task for the three different speller sizes.

Once the matrix of weights of the classifier was loaded to the system, the online task started. For the online task, the words and the number asked to spell were the following: “CHAT,” “PURE,” and “1935,” one after the other without spaces. They were instructed to continue without correcting the mistake in case a wrong letter was chosen by the classifier. Before each word's (or number's) set of flashes started, it was presented for 1 second on the screen (between seconds 2 and 3 of the 6 s pause). This time, the characters spelled appeared in a typing bar placed below the matrix of characters (Figure 2). The time required to choose a character was 36 s. Thus, the time taken to write the four characters of each word (or number) was 2 min and 24 s. The experimental design timing is shown in Figure 3.

**Figure 4:**
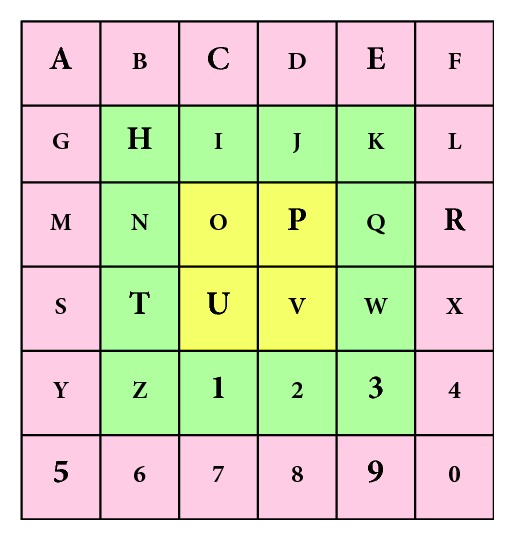
Distribution of the three layers according to their distance from the centre.

The choice of different characters during the online task was established so as to ensure that each target was located at different distances from the centre and at different directions. Thus, there are three layers in the 6 × 6 matrix from the edges to the middle (Figure 4). The three sequences of characters (two words and one number) to be spelt were selected so that each layer was covered by a similar percentage of characters (6/20 in layer 1, 4/12 in layer 2, and 2/4 in layer 3). Besides, in each layer, different characters were established so as to ensure that the user had to make, in the overt attention condition, gaze movements in different directions. The distribution of these characters is represented in bold in Figure 4.

After the online tasks, participants were asked to complete a visual analogue scale (VAS) of fatigue and the NASA-TLX test [41] and to answer a short questionnaire related to the speller tested in that session. This last questionnaire included three statements related to some features of speller size: (i) *statement 1*, difficulty perceiving different characters; (ii) *statement 2*, difficulty perceiving characters away from the centre; and (iii) *statement 3*, difficulty distinguishing different rows and columns. The participant expressed his/her level of agreement to each statement given a 10-point Likert scale (1 = very easy and 10 = very difficult).

The NASA-TLX test is a multidimensional rating questionnaire with six subscales (*mental demand*, *physical demand*, *temporal demand*, *performance*, *effort*, and *frustration*) which are scored between 0 and 100 and where higher values are related to higher levels of workload. This test consisted of two phases: In the first phase, participants give a rating to assign a magnitude to each subscale. In the second phase, 15 pairs of subscales were obtained after combining the six subscales, so the subjects could compare each pair to indicate and identify the subscale which affected their workload more. The overall workload was computed given a weighting average technique which considers the particular contribution of every subscale to the *total workload*. The overall workload values indicated the speller size requiring the most mental workload, while the weighted subscale scores identified the workload factors that made the greater contributions to each speller size. The highest possible score for the overall workload is 100, while the highest score for the weighted subscales is 33.3. The endpoints for each subscale are “very low/very high” except for the performance subscale, which has “perfect/failure” endpoints.

At the end of the third session, every subject was asked to compare the three speller sizes regarding his/her preferences. A comparative questionnaire adapted from the System Usability Scale (SUS) [42] allowed us to evaluate six dimensions: *favourite*, *complex*, *comfortable*, *stressful*, *controllable*, and *tiring*. For each dimension, the three speller sizes were ranked between them. Three ranks were proposed for each dimension: *rank 1*, the least; *rank 2*, intermediate; and *rank 3*, the most.

### 2.7. Parameter and Statistical Analysis

As mentioned in Introduction, the main objective of this study was to evaluate the usability of different speller sizes under two different conditions: covert and overt attention. The employed usability approach includes three dimensions: effectiveness, efficiency, and satisfaction. Effectiveness is related to the accuracy with which a user can complete tasks. In order to study the effectiveness, different results are required: (i) classification *accuracy* and *number of flashes* required to select a symbol during the calibration task; (ii) *error performance* in the online task writing all the words; and (iii) *amplitude* of the P300 signals during the online task. Efficiency is related to the resources expended to complete a task, i.e., user's effort and time required. In order to study the efficiency, the following metrics were provided: (i) the subjective workload assessed using NASA-TLX; (ii) the VAS of fatigue; and (iii) subjective questionnaires related to some features of speller size. Satisfaction is related to the users' attitude, i.e., the perceived comfort and acceptability while using the system. Results related to preference and subjective feelings regarding different speller sizes were analysed through the comparative questionnaire adapted from the SUS.  Table 2 summarises different objective and subjective metrics used to evaluate the three usability dimensions.

The analysis of variance (ANOVA) was used to analyse different evaluation metrics for effectiveness and efficiency dimensions. Additionally, multiple *R*-squared values were calculated for evaluating how well the model fits the data. Only results associated with a model with an *R*-squared value above 0.25 are reported here. A 5% threshold was considered significant for different Fisher's tests. Regarding the satisfaction dimension, in order to class the user's preference for each parameter (*favourite*, *complex*, *comfortable*, *stressful*, *controllable*, and *tiring*), Fisher's exact test has been used for each condition. The main reason to select Fisher's exact test instead of the *χ*^2^ test is the low sample size.

### 2.8. Gaze Direction Control

In order to validate the experiments carried out under the covert attention condition, it was necessary to verify that the subjects fixated his/her gaze on a yellow dot placed at the centre of the screen. To this end, the gaze data were analysed offline during the evaluation phase (i.e., the online task). The instructions demanded that subjects had to fixate the centre of the screen during the flash period for selecting a letter (30 s) and could gaze the target to locate it during the 6 s pause between flash periods. Due to participants having to spell 12 characters, the trial time period for one experiment was 432 s. Of this period, subjects had to gaze the centre of the screen for 360 s (30 s × 12 characters), enabling them to use the 6 s pause between flash periods to shift attention and gaze at the new character, that is, during 72 s (6 s × 12 period flashes) of the total trial time. In order to check if subjects gazed the centre of the screen, we obtained, for each subject and each speller size (i.e., for each trial time), the total time that the gaze data, in samples of 0.5 s, was at a distance of ±2° from the centre. Taking into account that subjects had to gaze the centre of the screen for 360 s, the percentage of time above or below this period was calculated for each trial. In case of gazing the centre of the screen for all the trial time (i.e., for 432 s), the percentage should be 20%. In case of gazing the centre of the screen for less than 360 s, the percentage should be negative. Finally, the percentage of time ranged from 8.16 to 17.78% (due to technical difficulties, gaze data were not available for two participants); that is, all the subjects gazed the centre of the screen for more than 360 s per trial. The average ± standard deviation (SD) percentages of all subjects were 12.68 ± 3.32, 13.19 ± 2.43, and 13.41 ± 2.53% for small, medium, and large speller sizes, respectively. These results show that the subjects followed the instructions; that is, they looked at the yellow dot under covert attention condition.

## 3. Results

### 3.1. Effectiveness Dimension

#### 3.1.1. Classification Accuracy during Calibration Task

**Figure 5:**
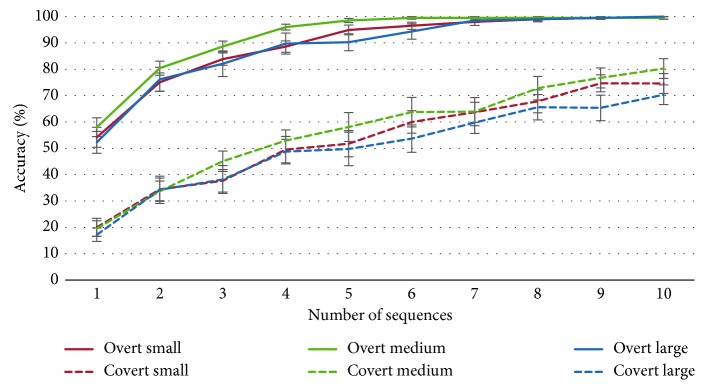
Average (±SE) of classification accuracy of the three speller sizes over the number of flash sequences for overt (solid line) and covert (dashed line) attention conditions.

Figure 5 shows the averages of the classification *accuracy* achieved by the participants, under both attentional conditions and the three tested speller sizes, which is related to the number of times that a row and a column in the matrix were intensified (i.e., the number of flashes, with one flash being the intensification of one row and one column). Regardless of the attentional condition or the speller size, the classification *accuracy* increases with the number of flashes.

In the overt attention condition, all subjects, except subject S4 who had 94% *accuracy* for small and medium speller sizes, obtained 100% of classification accuracy at the end of the ten flashes. In the covert attention condition, classification *accuracy* did not reach 100% for any subject (except for subjects S2, S3, and S9 using the medium speller size). In this condition, after 10 sequences (i.e., 20 flashes), the classification *accuracy* ranged from 63 to 94% for the small speller size, 63 to 100% for the intermediate speller size, and 50 to 94% for the large speller size, with averages (±standard error, SE) of 74.58 ± 13.09, 80.27 ± 12.95, and 70.33 ± 12.87% for small, medium, and large speller sizes, respectively.

**Figure 6:**
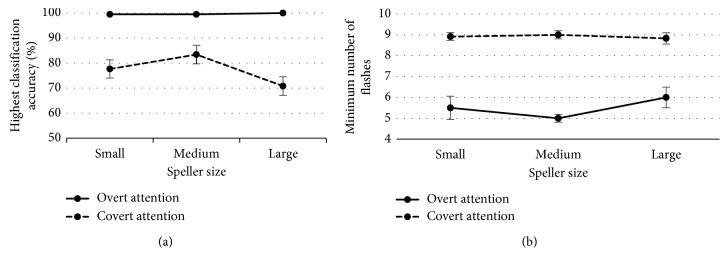
Mean (±SE) of the highest classification accuracy achieved by participant (a) and the minimum number of flashes required to reach that accuracy (b) during calibration task for each condition and each speller size.

For comparative results between speller sizes and attentional conditions, we calculated the average of the *highest classification accurac*y obtained during the calibration task and the *minimum number of flashes* required to obtain these accuracies. Results are depicted in Figures 6(a) and 6(b), respectively.

According to the *highest classification accuracy*, a two-way ANOVA (3 × 2) with factors *Speller Size* and *Attention* showed a significant interaction between these factors [*F* (2, 65) = 3.07; *p*=0.05]. In the overt attention condition, analysis showed no significant differences between speller sizes; however, in the covert attention condition, the mean *accuracy* for medium speller size (83.4 ± 3.7%) was the highest, offering significant differences versus the large speller size (70.8 ± 3.7%) (*p*=0.01). The ANOVA showed a significant main effect of factor *Attention* [*F* (1, 65) = 101; *p* < 0.001]. Independent of the speller size, the accuracy was significantly lower for the covert attention than for the overt attention condition. These results showed that calibration did not represent a problem under the overt attention condition, independent of the speller size. However, in the covert attention condition, *accuracy* did not reach 100% for any subject, which will affect the online performance. In spite of that, it is important to mention that medium speller size may have an influence on *highest classification accuracy*. Thus, the best classification accuracies were offered by the medium speller size.

According to the *minimum number of flashes* required to obtain the *highest classification accuracy*, the two-way ANOVA with factors *Speller Size* and *Attention* did not show any significant interaction between these factors. Thus, the differences in the *minimum number of flashes* required for each condition of *Attention* (i.e., overt and covert) between the factor *Size* (i.e., small, medium, and large conditions) were similar. Only the *Attention* factor was significant [*F* (1, 69) = 116; *p* < 0.001]. Therefore, independent of the speller size, the *minimum number of flashes* required was higher for covert attention than for overt attention. In the overt attention condition, the averages obtained for *minimum number of flashes* were 5.8 ± 0.5, 4.9 ± 0.2, and 5.8 ± 0.6 for the small, medium, and large sizes, respectively. However, most of participants (S1–S3, S6, S8–S10, and S12) required a lower *minimum number of flashes* to obtain 100% of *accuracy* with the medium speller size. In the covert attention condition, the averages for *minimum number of flashes* were 8.9 ± 0.3, 9.0 ± 0.3, and 8.8 ± 0.3 for the small, medium, and large sizes, respectively. However, as mentioned before, the accuracies did not reach 100%. It is important to notice that a reduced *minimum number of flashes* required to select a symbol would lessen the time to select it, increasing the efficiency of the system.

#### 3.1.2. Classification Accuracy during Online Task

**Figure 7:**
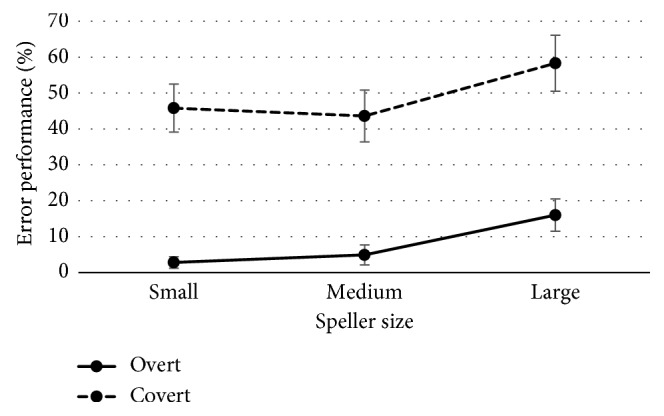
Average (±SE) error performance (%) by speller size in overt and covert attention modes during the online task.

Table 3 shows the overall performance results on the online task for each subject and each speller size. The “Mean” column represents the average ± standard error in *error performance* between speller sizes. Additionally, Figure 7 has been presented to visually observe the differences.

The *error performance*, calculated as the inverse of *accuracy* (i.e. 1 − *accuracy*) was analysed using a two-way ANOVA (3 × 2), with factors *Attention* and *Speller Size*. The obtained results did not show any significant interaction between these factors. However, a significant effect was obtained for *Attention* [*F* (1, 67) = 83.5; *p* < 0.001] and for *Speller Size* [*F* (2, 67) = 3.56; *p*=0.03]. Regarding the factor *Attention*, analysis revealed higher *error performances* for the covert attention than for the overt attention, independent of the speller sizes. Regarding the factor *Speller Size*, for both conditions, the *error performances* were significantly higher with the large speller size than for both the medium (*p*=0.02) and small speller sizes (*p*=0.02). Additionally, *error performances* were not different between the small and medium speller sizes. It is important to notice that, under the overt attention, the total number of subjects with 0% error was 9, 8, and 3 for the small, medium, and large sizes, respectively. However, the total number of subjects with more than 10% error was only 1 for the small and medium sizes and 5 for the large size. Otherwise, under the covert attention, only 2 participants achieved a 0% error performance (below 10%) and both using the same size, the large one.

To study the effect of the *Speller Size* and *Attention* factors on different layers of a speller (Table 4), three two-way ANOVA (3 × 2) have been carried out, one for each layer. First, the *Speller Size* factor has shown significant differences in *layer 1* [*F* (2, 67) = 4.4; *p*=0.01] and *layer 2* [*F* (2, 67) = 3.4; *p*=0.03]. On the one hand, for *layer 1*, we observed that the large size has obtained a worse performance compared to the medium size (*p*=0.01) and the small size (*p*=0.01). On the other hand, for *layer 2*, the same significant differences were obtained, with the large speller offering the highest percentage of *error performance* compared to the medium (*p*=0.01) and small (*p*=0.03) sizes. Second, in reference to *Attention* factor, the condition of covert attention has shown a significantly lower performance than the overt attention condition in all layers (*layer 1* [*F* (1, 67) = 81.7, *p* < 0.001], *layer 2* [*F* (1, 67) = 67.9, *p* < 0.001], and *layer 3* [*F* (1, 69) = 13.4, *p* < 0.001]). Finally, no interaction effect was found between the *Attention* factor and *Speller Size* in any layer.

#### 3.1.3. P300 Waveform Analysis

**Figure 8:**
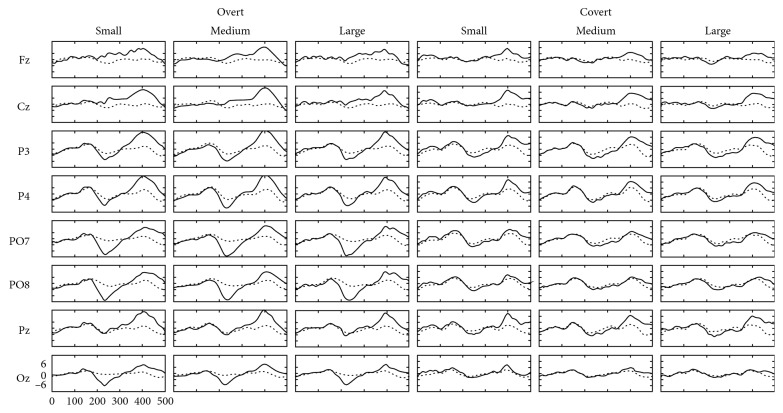
Grand average P300 waveforms (amplitude in *µ*V for *y* axis and time in ms for *x* axis) for target stimuli (solid) and nontarget stimuli (dashed) for the eight electrodes used (Fz, Cz, P3, P4, PO7, PO8, Pz, and Oz) as a function of the attention condition (overt and covert) and speller size (small, medium, and large).

Figure 8 shows the overall grand average event-related potential (ERP) waveforms obtained as a response to target and nontarget stimuli for the eight electrodes and as a function of the speller size for overt and covert attention conditions. For the three speller sizes and attentional conditions, different responses have positive peaks between 300 and 500 ms.

**Figure 9:**
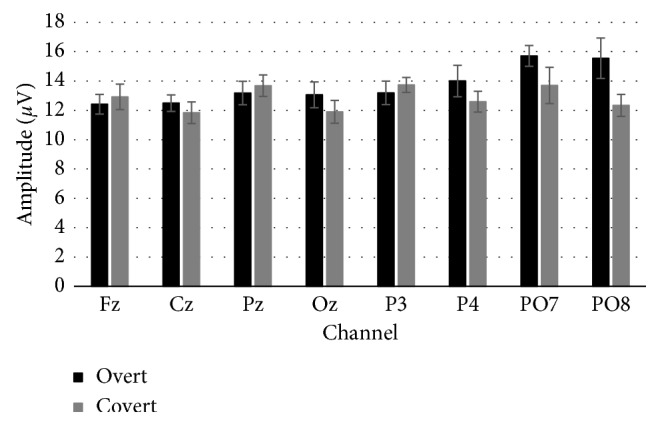
P300 peak amplitude for target and nontarget stimuli for each channel.

In order to investigate the effects of the overt versus covert attention and the speller size over the *latency* and *amplitude* of the P300, two three-way ANOVA (3 × 2 × 8), one for each dependent variable, were performed using the following factors: *Speller Size* (small, medium, and large), *Attention* (overt and covert), and *Channel* (Fz, Cz, Pz, Oz, P3, P4, PO7, and PO8). Regarding the *latency*, no main or interaction effects between factors were found. On the contrary, in reference to the *amplitude* of the P300, only an interaction effect of *Attention* × *Channel* [*F* (7, 77) = 3.456; *p*=0.023] was found. As such, paired *t*-test analysis for each channel comparing the *amplitude* between both overt and covert attentions (*Attention* factor) was carried out. These analyses have shown that significant differences have been restricted only to channels P4 [*t* (11) = 2.444; *p*=0.033] and PO8 [*t* (11) = 3.121; *p*=0.01], where the overt attention condition presented higher values (Figure 9).

### 3.2. Efficiency Dimension

#### 3.2.1. Workload and VAS Fatigue

Besides the NASA-TLX test, to evaluate the workload provoked by the use of the speller, overall fatigue was rated on a VAS ranging from 0 to 10 [43].  Table 5 shows the contributions of *VAS fatigue*, *total workload* (NASA-TLX global score ranged from 0 to 100), and dimensions to assess the subjective workload (*mental demand*, *physical demand*, *temporal demand*, *effort*, *performance*, and *frustration*, ranging from 0 to 33.3) for each speller size. The obtained values correspond to the average score among participants.

Another two-way ANOVA (3 × 2) was carried out in order to study the effect of *Speller Size* and *Attention* in the workload and fatigue. There was no main effect of the *Speller Size* or interaction effect between *Speller Size* and *Attention* in any variable. However, the workload and fatigue were generally higher for participants in the covert attention condition compared to the overt attention condition. Specifically, the covert attention condition offered significantly higher values for the following dimensions: *VAS fatigue* [*F* (1, 69) = 6.08; *p*=0.01], *total workload* (NASA-TLX) [*F* (1, 69) = 26.27; *p* < 0.001], *mental demand* [*F* (1, 69) = 6.98; *p*=0.01], *temporal demand* [*F* (1, 69) = 6.29; *p*=0.01], *effort* [*F* (1, 69) = 9.71; *p*=0.002], and *performance* [*F* (1, 69) = 6.03; *p*=0.01]. Interestingly, *physical demand* and *frustration* dimensions did not seem to be influenced by the *Attention* factor.

#### 3.2.2. Perception Subjective Questionnaires

The answers given by the participants at the end of each session in the usability questionnaire related to some features of the speller size are shown in Table 6. In this table, only the median of the answers in the sample of participants is given (ranging from 1 to 10: 1 = very easy and 10 = very difficult). Three two-way ANOVA were carried out to study the effects of *Speller Size* and *Attention* factors in different statements: *statement 1*, the difficulty perceiving different characters; *statement 2*, the difficulty perceiving characters away from the centre; and *statement 3*, the difficulty distinguishing different rows and columns.

The *Speller Size* factor showed significant differences in *statement 1* [*F* (2, 22) = 4.322; *p*=0.026] and *statement 3* [*F* (2, 22) = 5.204; *p*=0.014]. Thus, it could be affirmed that the *Speller Size* affects the difficulty perceiving different characters and distinguishing different rows and columns. Specifically, in *statement 1*, the medium speller size offered better values (i.e., lower scores in difficulty) than small (*p*=0.025) and large (*p*=0.019) speller sizes. On the contrary, for *statement 3*, the small speller size offered worse significant results than medium (*p*=0.023) and large sizes (*p*=0.048). Regarding the *Attention* factor, we found that the covert attention condition showed significantly higher scores, i.e., worse rating, for all statements (*statement 1* [*F* (1, 44) = 44.295; *p* < 0.001], *statement 2* [*F* (1, 11) = 88.393; *p* < 0.001], and *statement 3* [*F* (1, 11) = 44.044; *p* < 0.001]). In addition, an interaction effect was observed in *statement 2* between *Speller Size* and *Attention*. In order to study this interaction, two one-way ANOVA using the *Speller Size* factor were carried out for each attentional condition (i.e., overt and covert). No significant differences between speller sizes for the overt attention condition were found. However, there was significant differences between speller sizes under covert attention [*F* (2, 22) = 4.32; *p*=0.026], offering the medium speller size a lower difficulty perceiving characters away from the centre than the large one (*p*=0.017).

### 3.3. Satisfaction Dimension

Table 7 shows the usability questionnaire used for evaluating different dimensions concerning the participants' preference. Three ranks were proposed for each dimension: *rank 1*, the least; *rank 2*, intermediate; and *rank 3*, the most. The obtained results correspond to the subjects´ distribution according to the rank in function of the speller size for each dimension. To determine whether the users´ preference for speller size was significant, Fisher's exact test was conducted. The results relative to the overt attention condition were presented first, followed by the results relative to the covert attention condition. Finally, both attentional conditions were considered together to offer a general perspective.

#### 3.3.1. Overt Attention Condition

Regarding the result of overt attention, according to the test, no significant differences between speller sizes mobile for the *favourite* and *controllable* dimensions were obtained. However, Fisher's exact test showed a significant relation between the speller size and preference (order rank) for the following dimensions:

- Complex:
    50% of subjects chose the large speller size as the least *complex* (*rank 1*)
    75% of subjects chose the small speller size as the most *complex* (*rank 3*)
    50% of subjects chose the medium speller size as the intermediate (*rank 2*)
- Comfortable:
    67% of subjects chose the small speller size as the least *comfortable* (*rank 1*)
    42% of subjects chose the large speller size as the most *comfortable* (*rank 3*)
    75% of subjects chose the medium speller size as the intermediate (*rank 2*)
- Stressful:
    50% of subjects chose the small speller size as the least *stressful* (*rank 1*)
    50% of subjects chose also the small speller size as the most *stressful* (*rank 3*)
    67% of subjects chose the medium speller size as the intermediate (*rank 2*)
- Tiring:
    58% of subjects chose the large speller size as the least *tiring* (*rank 1*)
    75% of subjects chose the small speller size as the most *tiring* (*rank 3*)
    75% of subjects chose the medium speller size as the intermediate (*rank 2*)

In the overt attention condition, the following results could be observed: the small size was considered as the most *complex* and *tiring*, and the less *comfortable*; the medium size was classified as moderate, without presenting any negative score (i.e., the most *complex*, *stressful* or *tiring*, but the least *comfortable*); and the large size obtained the best value for *complex*, *comfortable*, and *tiring*. Additionally, it should be remarked that the medium size was the condition in which the users showed a greater agreement scoring it.

#### 3.3.2. Covert Attention Condition

In reference to the results relative to the covert attention condition, according to the test, no significant differences among speller sizes for the *complex*, *stressful*, and *controllable* dimensions were obtained. However, Fisher's exact test showed a significant relation between the speller sizes, under covert attention, and preference (order rank) for the following dimensions:

- Favourite:
    42% of subjects chose the small speller size as the least *favourite* (*rank 1*)
    50% of subjects chose the small and the medium speller sizes as the most *favourite* (*rank 3*)
    67% of subjects chose the large speller size as the intermediate (*rank 2*)
- Comfortable:
    58% of subjects chose the small speller size as the least *comfortable* (*rank 1*)
    33% of subjects chose the large or the small or the medium speller size as the most *comfortable* (*rank 3*)
    67% of subjects chose the medium speller size as the intermediate (*rank 2*)
- Tiring:
    42% of subjects chose the large speller size as the least *tiring* (*rank 1*)
    58% of subjects chose the small speller size as the most *tiring* (*rank 3*)
    67% of subjects chose the medium speller size as the intermediate (*rank 2*)

In general, under the covert attention condition, the following results have been obtained: the small size was considered as the most *favourite* (tied with the medium size) and the most *tiring*; the medium size was scored as the most *favourite* (tied with the small size); and the large size was considered as the least *tiring*.

#### 3.3.3. Overt and Covert Attention Conditions

**Figure 10:**
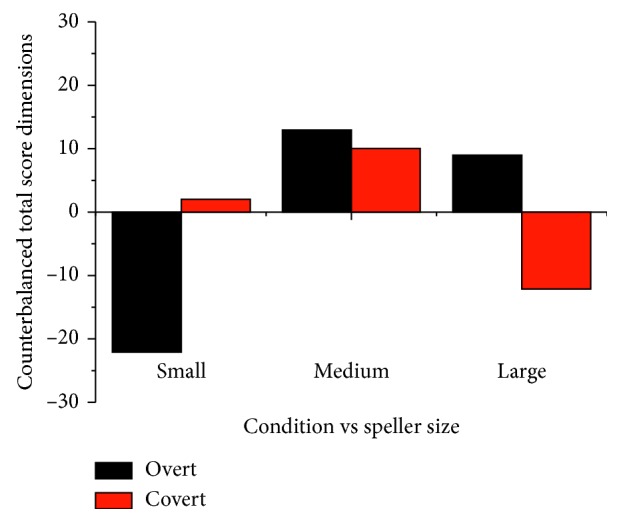
Representation of counterbalanced global dimensions to evaluate satisfaction of different interfaces. Rank was considered as a factor, which was multiplied by the subject's number that classified each dimension in ranks 1, 2, or 3. Each rank was translated in a positive or negative value (rank 1, ±1; rank 2, ±2; and rank 3, ±3). Positive values were assigned to *favourite*, *comfortable*, and *controllable* dimensions. Negative values were attributed to *complex*, *stressful*, and *tiring* dimensions.

To offer a global perspective, each variable of the satisfaction construct was classified as negative (*complex*, *stressful*, and *tiring*) or positive (*controllable*, *comfortable*, and *favourite*). Thanks to this classification, it can be affirmed that the small speller size presented more negative than positive dimensions since it was considered as very *complex,* not very *comfortable* and *tiring* (Figure 10). The large speller size obtained as much positive as negative dimensions because it was considered moderately *comfortable* and not very *tiring*. By contrast, most dimensions of the medium speller size presented mainly positive values, and it was classified largely in *rank 2*. Thus, in general, the medium speller size was chosen as enough satisfactory by users.

## 4. Discussion

In this study, the impact of speller sizes has been evaluated under constraint or nonconstraint conditions (i.e., covert and overt attention conditions, respectively) on both objective and subjective parameters. It has been shown that constraint to eye movement represents an important effort that is correlated with lower performance and higher workload. The usability measures suggested that medium speller size is the most convenient to ensure comfort and control using a visual P300-based speller.

Several studies are focused on defining optimal parameters to improve speller design in order to be more useful. Effects of matrix size [22, 26], interstimulus interval [22], luminosity contrast [24], and interface colour contrast [25] on different factors, such as P300 event-related potential or subjective measures, have been considered. The main objective of this research was to design BCI systems that are not only accurate but also easily usable for healthy subjects and, especially, for patients.

The effect of matrix size has already been studied. However, speller size has not been considered when designing a P300-based speller. Different speller sizes have been proposed in the literature but without knowing the effects they could have on usability. The inclusion of usability analysis at the early stage could be beneficial for the progression of the ALS's research.

In this study, three usability factors were evaluated: effectiveness, efficiency, and satisfaction [12]. The main objective was to determine which speller size obtained the best degree of usability according to the three dimensions under each attentional condition, overt and covert attention. This study has demonstrated that speller size has an important influence on user performance and must be considered when a BCI system is being designed. The results presented above are discussed in the following paragraphs.

### 4.1. Effect of Speller Size and Attention on Effectiveness Dimension

The present study has replicated the results of previous works, in which it was shown that performance in classification was severely impaired due to the lack of ocular mobility (i.e., under covert attention condition) [30, 33]. However, the effect of *Speller Size* factor had not adequately been studied previously; therefore, we will focus our discussion on this factor.

On the one hand, for the calibration task in the overt attention condition, there were no significant differences in classification *accuracy* between sizes (Figure 6(a)). On the other hand, for the covert attention, the medium speller size (83.4 ± 3.7%) seemed to show the best results, especially against the large size (70.8 ± 3.7%). These results could lead to the conclusion that the *Speller Size* factor is important to achieve a good *accuracy* in the case of users who do not have ocular mobility but less relevant when the user can control his/her eyes movements.

Regarding the *minimum number of flashes* required to achieve the maximum accuracy, it can be observed that more flashes were needed under covert attention (Figure 6(b)), presenting all sizes a similar *minimum number of flashes*. Under overt attention, all subjects obtained 100% *accuracy* before the 10 required flashes (one flash is the intensification of one row and one column), except for subject S4, who reached only 94% *accuracy* with the small and medium speller sizes. Contrastingly, under covert attention, the 100% *accuracy* was reached only by three participants (S2, S3, and S9) using the medium speller size.

In spite of having the possibility of reducing the time taken to select a letter during the online task (at least under overt attention), because the main objective of the study was to compare different speller sizes, we decided not to modify this parameter. For this reason, because the time required to select a symbol was always the same, the information transfer rate (ITR) to compare performances was not used. Nevertheless, it is important to notice that, under overt attention, a vast majority of subjects (8 subjects) was needed to calibrate the system with a reduced number of flashes when using the medium speller size. In this sense, the medium speller size under overt attention seems to require less number of flashes (4.9 ± 0.2 *minimum number of flashes*) and thus, less time to select a character. However, it was not the case for results obtained in constraint conditions where a higher number of flashes to obtain maximum classification performance were observed for all sizes (*minimum number of flashes*: small, 8.9 ± 0.3; medium, 9.0 ± 0.3; and large, 8.8 ± 0.3).

In reference to the performance in the online task, as expected, the *error performance* was affected by the attention condition (overt, 7.9%; covert, 49.3%). In addition, the *error performance* associated with the large speller size obtained the worst performance for both attentional conditions (Figure 7). In fact, the same trend was presented by overt and covert attention: the small (overt, 2.8 ± 1.6%; covert, 45.8 ± 6.7%) and medium (overt, 4.9 ± 2.8%; covert, 43.7 ± 7.2%) sizes have shown similar results, while the largest size offered the worst *error performance* regardless of *Attention* factor (overt, 16 ± 4.5%; covert, 58.3 ± 7.8%). In general, these results could lead us to think that there is a size from which the capacity to obtain an adequate *accuracy*/*error performance* decreases rapidly, i.e., the size between the medium and the large speller conditions.

In contrast with another study [29], in which participants had higher accuracy on a computer monitor than that on a mobile phone screen, the present work showed that the *error performance* was significantly higher for the large speller size compared to the small and medium speller sizes. In this sense, the worst performance is obtained when using the large speller size compared to the other two speller sizes. Effectively, except for participants S2 and S6, the remaining users obtained worse or equal error percentages when using the large speller. It is important to mention that, in the other study [29], the only information provided about different parameters of the matrix size was the visual angle of the screen, which was 3.7° and 3.56° for global positioning system (GPS) and cell phone screen, respectively. No information was provided regarding speller size, symbol size, or distance between columns and rows. Probably, these parameter values were lower than those used in our small speller size, making it very difficult to identify different characters. Otherwise, in our experiment, the smallest speller size was chosen according to the smallest symbol size so that it could be used without loss of performance. This minimum size was reported in a different study [28]. Therein, symbol sizes would decrease the performance considerably, being probably the reason of the low performance obtained with the study on mobile phone and the GPS screens [29].

Sellers at al. [22] have reported that matrix size, i.e., the number of elements, has a significant effect on performance: a 3 × 3 matrix offered better results than a 6 × 6 matrix. In the present work, the results obtained suggest that speller size can also have a significant effect on user performance, and thus it is an important factor to consider it in the design. The obtained results show how *error performance* increases when using the large speller size, which has frequently been used by other researchers (e.g., [30, 33]). The best performances were obtained when using the small and medium speller sizes, achieving similar performance.

The analysis of layers in the online task, which is closely related to the size, has shown how the *Attention* factor presented an effect on all layers, not only on the outer ones. These results show the importance of ocular mobility, even when the stimuli are close to the point of view. On the contrary, the *Speller Size* factor has exclusively influenced *layer 1* and *layer 2*, the two most external layers. Specifically, in these layers (*layer 1* and *layer 2*), we observed that the large size showed a worse performance than the medium and small sizes. Thus, the worst combination is the large size in the external layers, that is, where the character is placed at the furthest position from the centre of the screen.

Ultimately, it seems clear that the large size is associated with a worse performance, especially under covert attention. Therefore, it should be recommended to avoid the large size in order to achieve a good level of *accuracy*, especially in the case of patients with impaired ocular mobility.

Finally, in reference to the analysis of the P300 signal, while matrix size affects P300 peak amplitude due to different target probabilities according to different matrices [22, 26], the obtained results showed no significant main effects on P300 response for speller size. Moreover, because the three speller sizes have the same matrix size (6 × 6), different P300 amplitudes for the target and nontarget conditions were similar for different speller sizes. Similar conclusions were obtained in another study [28]. However, the attention factor showed significant differences in amplitude levels of the target stimulus at channels P4 (overt, 14 ± 1.07 *µ*V; covert, 12.59 ± 0.71 *µ*V) and PO8 (overt, 15.55 ± 1.38 *µ*V; covert, 12.34 ± 0.75 *µ*V). Thus, it has been corroborated that the performance differences previously presented between covert and overt attention conditions have a neural correlate [30].

### 4.2. Effect of Speller Size and Attention on Efficiency Dimension

In the present work, the fatigue and workload to efficiency assessments have been studied, including in the workload are the following dimensions: *total workload*, *mental demand*, *physical demand*, *temporal demand*, *effort*, *performance*, and *frustration*. Results showed that the effect of *Attention* factor has been important since it has shown significant differences in all measured dimensions, with the exception of *physical demand* and *frustration*. On the contrary, although the ANOVA did not show significant differences for the *Speller Size*, it can be observed in Table 5 that the medium speller has shown the lowest *total workload* score for both conditions, i.e., overt and covert attention conditions.

Regarding the questions related to perception (*statement 1*, *statement 2* and *statement 3*), it should be noted that the medium speller size has obtained the most appropriate scores for each statement in both conditions of the *Attention* factor (Table 6). In the same way, it can be observed how the scores under the covert attention condition have been higher for each of different speller sizes used. Therefore, it seems clear that the inability to move the eyes significantly affects the necessary cognitive resources used to control the interface. This disability should be considered in the case of several patients who cannot perform such action by using interfaces with an adequate size, since the medium size presented the best results despite the lack of significances, or new features that reduce the cognitive resources needed to use the speller (e.g., [44]).

In the previously mentioned work [29], subjects reported that smaller screens (GPS and especially mobile phone screens) were too difficult to read due to the difficulty to perceive the target symbol. However, the varying results between that work and our study could be explained by the differences in the employed sizes or other characteristics of the interface, such as the screen resolution.

In reference to the difficulty perceiving the presented stimuli (i.e., *statement 1*, *statement 2*, and *statement 3*), thanks to the related results with the *Attention* factor, it could be concluded that the inability to move the eyes is a key factor that provokes a significant impairment in the facility to perform the task. Regarding the speller size, the medium speller presented the lowest difficulty perceiving the characters (*statement 1*) in contrast to small and large spellers, while the small size speller showed the worst results in difficulty distinguishing different row and columns (*statement 3*) in contrast to medium and large spellers. Thus, the medium speller size was always related to the best, or lower, scores in difficulty since no other speller size obtained better significant results versus it. Additionally, the interaction effect between *Speller Size* and *Attention* in *statement 2* (the medium speller size was better than the large one under covert attention, but not under overt attention) shows that the size is not important under the overt attention condition. This factor should instead be considered under covert attention.

### 4.3. Effect of Speller Size and Attention on Satisfaction Dimension

Satisfaction has been studied according to six dimensions: *favourite*, *complex*, *comfortable*, *stressful*, *controllable*, and *tiring*. For each dimension, the participants had to rank the three speller sizes. On the one hand, the most remarkable point regarding the overt attention condition was that, according to Table 7, the small speller size was the worst rated of all, while the medium and large speller sizes obtained the best scores. On the other hand, for the covert attention condition, the large speller was the worst valued in general, with the medium speller being the best valued. Therefore, three conclusions could be obtained: (i) the small speller was the worst rated in the overt attention condition, (ii) the large speller was the worst rated in the covert condition, and (iii) the medium speller has obtained the best rating in both conditions. Thus, as it was depicted in Figure 10, the medium speller could be denoted as the most satisfactory, regardless of the condition, since it has shown a tendency to offer the best results.

## 5. Conclusions

The present work has investigated the usability of three speller sizes under overt and covert attention handling a P300-based BCI speller. The obtained results showed that, in both attentional conditions (i.e. overt and covert attention), the speller size had significant effects, or trends that should not be ignored, on user usability considering the effectiveness, efficacy, and satisfaction. Regarding the effectiveness, the large speller size offered the worst results under overt and covert attention, while medium and small offered similar results, with a slight superiority of the medium size. In reference to efficacy, the large speller size offered a trend in which it gathered the worst values according to different NASA-TLX workload measures and fatigue. Finally, regarding the satisfaction dimension, the medium speller size was the best rated, while the larger speller size obtained the worst general results under covert attention because the gaze movements were restricted and the distance between symbols was larger. Additionally, the small speller size offered the worst results under overt attention, due perhaps to the denoted tiredness provoked by this size. Therefore, based on the trends offered by the medium speller size and the lack of worst obtained results when using it among all usability dimensions, this size may be the most recommended to employ.

In short, we have demonstrated that the speller size should be considered in the usability of a P300-based BCI speller, although it may also depend on whether or not the participant has the ability of gaze control. For future works, we recommend to continue the study relative to the speller size to be able to confirm the present results found. Some examples of possible studies include testing other sizes, such as those under our small speller size or the size in which the performance in *accuracy*/*error performance* was significantly decreased between the medium and large speller sizes. In addition, in order to fulfill this aim, it could be convenient to increase the sample size and the number of letters written in the calibration and online tasks, as well as to assess this effect of the speller sizes in patients, instead of able-bodied participants, under the covert attention condition.

## Figures and Tables

**Table 1:** Different parameters used in different speller sizes.

Parameters		Size		
		Small	Medium	Large
Matrix size (MS)	cm	5.27	9.98	14.69
	inch	2.26	3.92	5.78
Symbol size (SS)	cm	0.42	0.79	1.17
	inch	0.16	0.31	0.46
Symbol distance (SD)	cm	0.55	1.04	1.53
	inch	0.22	0.41	0.61

**Table 2:** Different evaluation metrics used to analyse the three usability dimensions.

Effectiveness	(i) Classification accuracy and number of flashes required to select a symbol during calibration
	(ii) Error performance according to the online task
	(iii) P300 waveforms (amplitude and latency)
Efficiency	(i) NASA-TLX
	(ii) VAS fatigue
	(iii) Perception subjective questionnaires
Satisfaction	(i) A comparative questionnaire adapted from the SUS for six dimensions (favourite, complex, comfortable, stressful, controllable, and tiring)

**Table 3:** Error performance (%, mean ± SE) results of the online task for each participant.

Participant	Overt attention				Covert attention			
	Small	Medium	Large	Mean	Small	Medium	Large	Mean
S1	16.7	0	41.7	19.4 ± 12.1	66.7	33.3	66.7	55.6 ± 11.1
S2	8.3	0	0	2.8 ± 2.7	83.3	41.7	50	58.3 ± 12.7
S3	0	0	41.7	13.9 ± 13.9	16.7	16.7	8.3	13.9 ± 2.8
S4	0	8.3	8.3	5.6 ± 2.8	16.7	66.7	41.7	41.7 ± 14.4
S5	0	0	8.3	2.78 ± 2.8	50	25	33.3	36.1 ± 7.4
S6	0	33.3	0	11.1 ± 11.1	41.7	75	100	72.2 ± 16.9
S7	0	0	16.7	5.6 ± 5.6	75	58.3	66.7	66.7 ± 4.8
S8	0	0	0	0 ± 0	25	25	33.3	27.8 ± 2.8
S9	0	8.3	25	11.1 ± 7.4	25	25	100	50 ± 25
S10	8.3	0	33.3	13.9 ± 10	33.3	75	58.3	55.5 ± 12.1
S11	0	8.3	8.3	5.6 ± 2.8	50	75	75	66.7 ± 8.3
S12	0	0	8.3	2.8 ± 2.8	66.7	8.3	66.7	47.2 ± 19.5
Mean	2.8 ± 1.6	4.9 ± 2.8	16.0 ± 4.5	7.87	45.83 ± 6.7	43.75 ± 7.2	58.33 ± 7.8	49.3

**Table 4:** Error performance (%, mean ± SE) results according to different layers of the matrix.

Layer	Overt attention				Covert attention			
	Small	Medium	Large	Mean	Small	Medium	Large	Mean
Layer 1 (red)	2.8 ± 1.9	6.9 ± 4.3	16.7 ± 4.6	8.8 ± 4.1	50 ± 7.1	44.5 ± 8.5	66.7 ± 7.7	53.7 ± 6.7
Layer 2 (green)	2.1 ± 2.1	2.1 ± 2.1	12.5 ± 5.8	5.6 ± 3.5	49.3 ± 9.1	45.8 ± 10.6	70.8 ± 9.7	55.3 ± 7.8
Layer 3 (yellow)	4.2 ± 4.2	4.2 ± 4.2	20.8 ± 9.7	9.7 ± 5.5	29.2 ± 9.7	41.7 ± 8.3	33.3 ± 11.2	34.7 ± 3.7

**Table 5:** VAS fatigue and NASA-TLX scores (mean ± SE), including six different dimensions such as mental demand, physical demand, temporal demand, effort, performance, and frustration.

Parameters	Overt attention				Covert attention			
	Small	Medium	Large	Mean	Small	Medium	Large	Mean
VAS fatigue	4 ± 0.6	2.8 ± 0.7	4.5 ± 1	3.8 ± 0.5	5.42 ± 0.7	4.8 ± 0.7	5.3 ± 0.8	5.2 ± 0.2
Total workload	40.4 ± 7.2	38.22 ± 4.8	41.2 ± 6.4	39.9 ± 0.9	65.1 ± 5.4	60.4 ± 6.4	66.9 ± 4.9	64.1 ± 1.9
Mental demand	9.9 ± 2.8	11.7 ± 2.9	12.5 ± 2.7	11.4 ± 0.8	16.7 ± 2.6	16.8 ± 2.7	18.0 ± 3	17.2 ± 0.4
Physical demand	3.8 ± 2.1	4.2 ± 1.7	6.3 ± 2.3	4.8 ± 0.8	6.9 ± 2.7	3.31 ± 1.1	6.39 ± 1.9	5.5 ± 1.1
Temporal demand	7.3 ± 1.3	9.2 ± 1.8	6.8 ± 1.8	7.8 ± 0.7	13.2 ± 2.9	9.6 ± 1.9	13.4 ± 2.4	12.1 ± 1.2
Effort	9.5 ± 2.7	7.9 ± 1.7	7.5 ± 1.8	8.3 ± 0.6	13.3 ± 2.3	13.4 ± 2.1	14.7 ± 2.4	13.8 ± 0.4
Performance	4.8 ± 1.8	3.8 ± 1.6	8.6 ± 2.7	5.7 ± 1.5	10.1 ± 1.8	13.6 ± 3.3	7.61 ± 1.9	10.4 ± 1.7
Frustration	4.8 ± 2.3	1.2 ± 0.7	3.5 ± 1.2	3.2 ± 1.1	4.95 ± 2.5	3.3 ± 1.1	6.78 ± 2.0	5.01 ± 1

**Table 6:** Average scores (±SE) of answers to the usability questionnaire.

Statements	Overt attention			Covert attention		
	Small	Medium	Large	Small	Medium	Large
Statement 1: difficulty perceiving different characters	2.4 ± 0.8	1.3 ± 0.3	1.8 ± 0.5	5.75 ± 0.7	4 ± 0.4	5.75 ± 0.5
Statement 2: difficulty perceiving characters away from the centre	2.2 ± 0.7	1.2 ± 0.4	1.8 ± 0.5	6.5 ± 0.6	5.9 ± 0.5	7.8 ± 0.4
Statement 3: difficulty distinguishing different rows and columns	2.7 ± 0.8	1.5 ± 0.4	1.6 ± 0.5	6.8 ± 0.7	5 ± 0.7	5.8 ± 0.8

**Table 7:** Subjects' distribution for each dimension.

Dimension	Overt attention				Statistics (Fisher's exact test)	Covert attention			Statistics (Fisher's exact test)
	Rank	Small	Medium	Large		Small	Medium	Large	
Favourite	1	6	3	3	*F* = 6.337; *p*=0.182	5 (42%)	3 (25%)	4 (33%)	*F* = 13.715; *p* = 0.006
	2	2	3	7		1 (8%)	3 (25%)	8 67%)	
	3	4	6	2		6 (50%)	6 (50%)	0 (0%)	
Complex	1	1 (8%)	5 (42%)	6 (50%)	*F* = 13.715; *p* = 0.006	5	5	2	*F* = 4.042; *p*=0.403
	2	2 (17%)	6 (50%)	4 (33%)		3	5	4	
	3	9 (75%)	1 (8%)	2 (17%)		4	2	6	
Comfortable	1	8 (67%)	0 (0%)	4 (33%)	*F* = 19.358; *p* < 0.001	7 (58%)	0 (0%)	5 (42%)	*F* = 13.577; *p* = 0.007
	2	0 (0%)	9 (75%)	3 (25%)		1 (8%)	8 (67%)	3 (25%)	
	3	4 (33%)	3 (25%)	5 (42%)		4 (33%)	4 (33%)	4 (33%)	
Stressful	1	6 (50%)	1 (8%)	5 (42%)	*F* = 13.715; *p* = 0.006	7	3	2	*F* = 7.359; *p*=0.115
	2	0 (0%)	8 (67%)	4 (33%)		1	6	5	
	3	6 (50%)	3 (25%)	3 (25%)		4	3	5	
Controllable	1	6	2	4	*F* = 6.24; *p*=0.205	4	4	4	*F* = 6.913; *p*=0.161
	2	1	6	5		1	6	5	
	3	5	4	3		7	2	3	
Tiring	1	2 (17%)	3 (25%)	7 (58%)	*F* = 21.288; *p* < 0.001	4 (33%)	3 (25%)	5 (42%)	*F* = 10.804; *p* = 0.033
	2	1 (8%)	9 (75%)	2 (17%)		1 (8%)	8 (67%)	3 (25%)	
	3	9 (75%)	0 (0%)	3 (25%)		7 (58%)	1 (8%)	4 (33%)	

Percentages are indicated for those dimensions with significant differences. Ranks are ordered as follows: *rank 1*, the least; *rank 2*, intermediate; and *rank 3*, the most. Significant results have been denoted in bold.

## Data Availability

The data used to support the findings of this study are available from the corresponding author upon request.
